# Supplementary material for: Identification of photoperiod-induced specific miRNAs in the adrenal glands of Sunite sheep (Ovis aries)
Source: Front Vet Sci. 2022 Jul 22;9:888207. doi: 10.3389/fvets.2022.888207 (PMC9354845; doi:10.3389/fvets.2022.888207)
Supplement: Supplementary file 1 [file Data_Sheet_1.ZIP › Supplementary file/miRDeep2 file.zip!/miRDeep2 file/Know_Structure/SP42A1/oar-mir-494.pdf]

miRBase precursor : oar-mir-494
Total read count : 15231
oar-miR-494-5p read count : 31
oar-miR-494-3p read count : 15199
remaining reads : 1

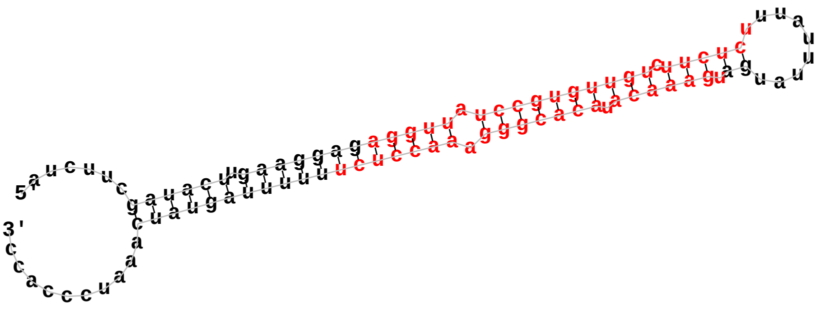


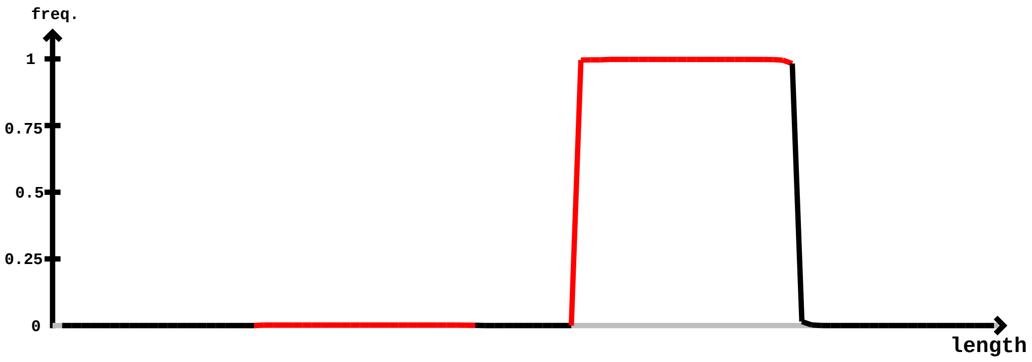


```
                                                                oar-miR-494-3p
                    oar-miR-494-5p
5'- aucuucgauacuugaaggagagguuauccguguugucuucucuuuauuuaugaugaaacauacacgggaaaccucuuuuuuaguaucaaaucccacc  -3'   exp
    ......((((((.((((((((((((.((((((((((.(((((.........)).)))))).))))))).))))))))))))))))))..........   reads      mm        sample
    ....................agguuauccguguugucuucu........................................................   10         0         seq
    ....................agguuauccguguugucuucuc.......................................................   5          0         seq
    ....................agguuauccguguugucuucucu......................................................   16         0         seq
    .............................................auuuaugaugaGacaua...................................   1          1         seq
    .....................................................ugaaacauacacgggaaac.........................   4          0         seq
    .....................................................ugaaacauacacgggaaacc........................   4          0         seq
    .....................................................ugaaacauacacgggaaaccu.......................   35         0         seq
    .....................................................ugaaacauacacgggaaaccuc......................   191        0         seq
    .....................................................ugaaacauacacgggaaaccucu.....................   14727      0         seq
    .....................................................ugaaacauacacgggaaaccucuu....................   191        0         seq
    .....................................................ugaaacauacacgggaaaccucuuu...................   12         0         seq
    .....................................................ugaaacauacacgggaaaccucuuuu..................   1          0         seq
    ......................................................gaaacauacacgggaaaccucu.....................   9          0         seq
    ........................................................aacauacacgggaaaccucu.....................   1          0         seq
    ........................................................aacauacacgggaaaccucuu....................   22         0         seq
    ........................................................aacauacacgggaaaccucuuuu..................   2          0         seq
```
